# Machine Learning Advances in Microbiology: A Review of Methods and Applications

**DOI:** 10.3389/fmicb.2022.925454

**Published:** 2022-05-26

**Authors:** Yiru Jiang, Jing Luo, Danqing Huang, Ya Liu, Dan-dan Li

**Affiliations:** State Key Laboratory of Microbial Technology, Institute of Microbial Technology, Shandong University, Qingdao, China

**Keywords:** microorganisms, machine learning, deep learning, prediction, classification

## Abstract

Microorganisms play an important role in natural material and elemental cycles. Many common and general biology research techniques rely on microorganisms. Machine learning has been gradually integrated with multiple fields of study. Machine learning, including deep learning, aims to use mathematical insights to optimize variational functions to aid microbiology using various types of available data to help humans organize and apply collective knowledge of various research objects in a systematic and scaled manner. Classification and prediction have become the main achievements in the development of microbial community research in the direction of computational biology. This review summarizes the application and development of machine learning and deep learning in the field of microbiology and shows and compares the advantages and disadvantages of different algorithm tools in four fields: microbiome and taxonomy, microbial ecology, pathogen and epidemiology, and drug discovery.

## Introduction

Microbiology focuses on studying the activity law of microorganisms, exploring the characteristics, culture conditions, and detection methods of microflora, taking its essence (discovering, utilizing, improving, and protecting beneficial microorganisms), and removing its dross (preventing, controlling, or transforming harmful microorganisms). Thus, it is available for science and benefits mankind (Dworkin, 2012; Hanage, 2014; Ha and Devkota, 2020).

Recently, the main research hotspots in microbiology include community classification and its environmental role (Bulgarelli et al., 2013; Zhang et al., 2021), regulation of gut microbiome and host interactions (Turnbaugh et al., 2007; Jones et al., 2014; Malla et al., 2018; Ruff et al., 2020), development of pathogenic microorganisms and drug vaccines (Shahbaaz et al., 2016; Moos et al., 2017; Zhu et al., 2020), and trying to dilute the boundaries between microbiome and genome editing, molecular modification, ecology and resource utilization, biocatalysis, and synthesis (Stres and Kronegger, 2019; Galloway-Pena and Hanson, 2020). In addition, microbiology and multiomics (including genomics, epigenomics, transcriptomics, proteomics, and metabolomics) have combined and developed a variety of multiscale emerging fields (Beck et al., 2021; Liang et al., 2021).

The understanding of microorganisms started from microbial cell morphology and physiological and biochemical characteristics to microbial genotype identification at the nucleic acid and protein levels, and chemical analysis methods based on cell chemical composition analysis and numerical classification methods relying on the level of computational biology have also been established successively. The rapid progress in the discipline of microbiology is inseparable from the update of observation methods or techniques in the same period (Galloway-Pena and Hanson, 2020). With the advent of the Big Data era, the pressing questions for researchers have gradually evolved into how to quickly and efficiently filter/condense this exponential growth of information to obtain generalized quality data and how to transform the massive data of microbiota into easily understood and visualized knowledge. Compared to traditional research with insufficient data or purely experimental techniques that cause trouble, such as cognitive bias, low reproducibility, and long-time span, the modern microbiology research process is more likely to incorporate new technologies and big data methods to do this better and right.

Artificial intelligence (AI), first proposed by John McCarthy at the Dartmouth Conference in the summer of 1956, concentrates on the simulation of human intelligence extensions and the research and development of theoretical methods, techniques, and applied systems. The entry of AI drives the progress of microbiology and achieves a new paradigm breakthrough (Barredo Arrieta et al., 2020). Combined with the advantages of big data, automation, modeling, and AI, microbiology has evolved toward a multiscale and multidimensional direction, gradually applying to systems biomedicine, systems ecology, etc.

Machine learning (ML), first proposed by Arthur Samuel (Bell Labs, IBM, Stanford) in 1959, is a special branch/subfield of AI that aims to find features from large-scale heterogeneous data. The most basic thing is to use algorithms to parse the data, analyze the patterns in the data automatically, and then utilize these patterns to make predictions and decisions on real-world events (Jordan and Mitchell, 2015). Unlike traditional software programs that are hard-coded to solve specific tasks, ML takes large amounts of data and trains them using algorithms to learn how to accomplish tasks from the data (Domingos, 2012). With the integration of cross-scale and complex microbial communities and multiomics integration, ML can be used to systematically present interactions between microflora or with hosts. The workflow of dimensionality reduction and then extraction of spatial features from high-dimensional datasets generated from large data collections is supportive of exploring the functional potential of microorganisms and expanding the study of microbial technology applications.

**Figure 1:**
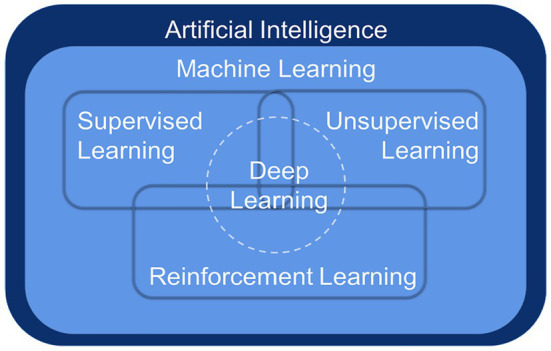
The relationship among artificial intelligence, machine learning, and deep learning.

Deep learning (DL) is a breakthrough ML approach that models high-level abstractions of data through a deep network with multiple layers of processing units, which are parametric models trained by gradient descent (Lecun et al., 2015). ML is a way to implement AI, and DL is a technology to implement ML (Figure 1). Remarkably, there is no obvious boundary separating DL from traditional ML and traditional statistical analysis. To handle complex, high-dimensional microbiome data, ML algorithms have been applied to the frontiers of combining microbiome and computational science, more commonly for classification and prediction (Schmidhuber, 2015).

This paper first briefly introduces the ML methods, data processing steps, and algorithms commonly used in microbial research, summarizes the research on ML-based microbial prediction and application, and discusses the advantages and limitations of the methods and tools, demonstrating the development prospects of computational microbiology from the perspective of ML.

## Machine Learning

**Figure 2:**
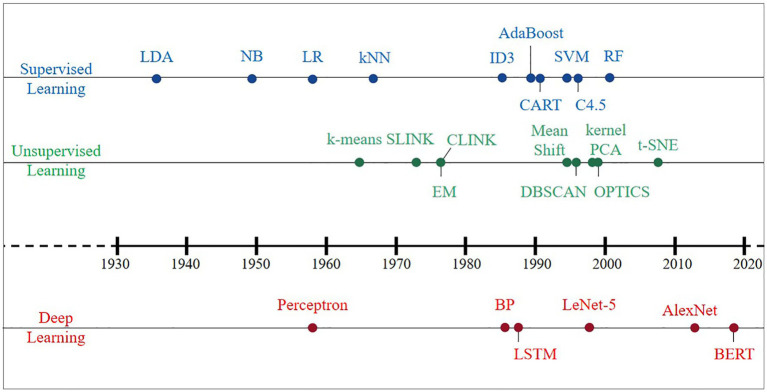
Development history of classical machine learning algorithms since the 1930s.

An AI system is supposed to be equipped to learn knowledge from raw data, which is known as ML. Effective features are extracted from raw data by designing targeted pattern recognition algorithms and then using these features with ML algorithms, i.e., distance functions to represent pairwise relationships between objects. The earliest ML algorithms can be traced back to the early 20th century, and a large number of classical methods have been developed within these 100 years (Figure 2). This section summarizes the classical algorithms that have appeared in history in four directions: supervised learning, unsupervised learning, DL, and reinforcement learning (RL). Then, we elaborate on the criteria for evaluating the merits of the model and algorithmic workflows.

### Supervised Learning

Supervised learning, including regression analysis and statistical classification, refers to a class of methods that use samples from known categories as training sets to train models. Before the concept of ML was introduced, Fisher (1936) invented a supervised data dimensionality reduction algorithm, linear discriminant analysis (LDA). In the 1950s, based on the core idea of Bayes decision theory, which is to select the decision with the highest probability, the Bayes classifier was born and divides the sample into the class with the highest posterior probability. The naive Bayes (NB) model has a simple algorithm with stable classification efficiency, performs well for small-scale data, can handle multiple classification tasks and is suitable for incremental training (Zhang et al., 2009); however, it is required to decide the probability of the posterior by virtue of the prior and data before classification determination. Thus, there is a certain error rate in the classification decision-making, and it is sensitive to the expression of the input data. Logistic regression (LR) directly predicts the probability of a sample belonging to a positive sample, with a clear model, strong parameter interpretability, and simple and efficient for big data scenarios; however, its performance is easily affected by the correlation between features and the size of the feature space, and it is prone to underfitting problems, resulting in low accuracy (Cox, 1958). The k-nearest neighbor (kNN) algorithm is considered an algorithm based on the idea of template matching that is simple and efficient and can solve both classification and regression problems with high accuracy and insensitivity to outliers; however, its prediction speed is slower than that of LR, especially for dealing with high-dimensional data, which is computationally intensive (Cover and Hart, 1967).

The year 1980 serves as a transition point in the history of ML algorithms, which gradually developed from fragmented and unsystematic enlightenment algorithms into an independent and systematic direction. Various machine learning algorithms have exploded and developed rapidly. In the 1980s and early 1990s, three typical implementations of decision trees (DT): ID3 (Quinlan, 1986), CART (Yeh, 1991), and C4.5 (Quinlan, 1996), had fast computation, high accuracy, and high interpretability, which make DT still used in some problems today, but their characteristic of easy-overfitting leads to easy neglect of the relevance of attributes in the dataset. Two classical algorithms, support vector machine (SVM) based on statistical learning theory (Cortes and Vapnik, 1995) and AdaBoost (Freund, 1990), were developed in the 1990s. The former (SVM) uses kernel functions that can be mapped to a high-dimensional space to solve nonlinear classification problems with uncomplicated classification ideas (maximizing the interval between samples and decision surfaces) and presents better classification performance; however, the method is difficult to solve the multiclassification problem, sensitive to missing data, and thus challenging to achieve large-scale training samples. The latter (AdaBoost) can integrate the use of simple weak classifiers, which does not require either *a priori* knowledge of weak classifiers or filtering of features, and can significantly improve learning accuracy regardless of whether the data are artificial or real; nevertheless, it is susceptible to noise interference and has a long training time.

The random forest (RF) and AdaBoost algorithms belong to integrated learning, with high accuracy, and can effectively run on large datasets and strong resistance to noise (Breiman, 2001); however, the number of decision trees will lead to a very long training time, and overfitting occurs in noisy classification or regression problems. Up until the rise of DL in 2012, supervised learning was rapidly developed, and various ideas and methods emerged one after another, yet no one ML algorithm achieved an overwhelming advantage.

### Unsupervised Learning

Unsupervised learning is a method to learn the commonality in the input data to determine whether such commonality exists in the new data, and the research thinking can be divided into two categories: clustering and data dimensionality reduction. The hierarchical clustering algorithm emerged early (Ward, 1963), and some of its implementations are still in use today, including SLINK (Sibson, 1973) and CLINK (Defays, 1977). The K-means clustering algorithm was then born, and the algorithm is simple and easy to implement (Macqueen, 1965), whereas there are the following drawbacks: (1) the number of class clusters needs to be specified by the user in advance; (2) the clustering results are more sensitive to the selection of the initial class cluster centers; (3) it is easy to fall into a local optimum; and (4) only spherical class clusters can be found; since then, it has been continuously improved and grows into the clustering algorithm with the most variants and improvements. The expectation-maximum (EM) algorithm (Dempster et al., 1977) has been used to solve various extreme likelihood estimation problems in ML with missing data and is commonly used to learn the variational inference of LDA topic models, parameters of the Gaussian mixture model (GMM), and hidden Markov model (HMM). Other density-based clustering algorithms in the 1990s include mean shift (Cheng, 1995), density-based spatial clustering of applications with noise (DBSCAN) algorithm (Ester et al., 1996), and ordering points to identify the clustering structure (OPTICS) algorithm (Ankerst et al., 1999). They are not based on various distances but on density.

A new idea of clustering was born in the early 21st century: transforming the clustering problem into the graph cutting problem, and the representative algorithm covering this new idea is spectral clustering. The data dimension reduction algorithm originated very early, and the advantages of the classic principal component analysis (PCA) algorithm are the complete absence of parameter restrictions, the removal of data redundancy and noise, the compression and preprocessing of the data to make the dataset easier to use, and the results easier to understand (Pearson, 1901). PCA can eliminate the correlation between variables, but the nonlinear dependence between samples may be lost if linear dimensionality reduction is performed *via* PCA. The heavyweight result innovation, kernel PCA (Scholkopf et al., 1998), was based on the kernel technique, combined with PCA and transforming PCA into a nonlinear dimensionality reduction algorithm. Since then, a wave of nonlinear methods has been set in motion, e.g., locally linear embedding (LLE), Laplacian eigenmaps, locality preserving projections, and isometric mapping (Roweis and Saul, 2000; Tenenbaum et al., 2000; Belkin and Niyogi, 2003; He and Niyogi, 2003). Then, *t*-distributed stochastic neighbor embedding (t-SNE) was developed (Van Der Maaten and Hinton, 2008), mainly for visualizing and exploring high-dimensional data, which follows nonlinearity and has the best visualization effect compared with other dimensionality reduction algorithms. The relative similarity of the original data at the time of dimensionality reduction is excellent; however, the results of each run will change slightly for each run due to its random nature. Unsupervised learning, although relatively slow in development and with few breakthroughs, has occupied a dominant role in human and animal learning and is a necessary path to explore strong artificial intelligence.

### Deep Learning

Deep learning, compared to traditional ML, is more highly dimensional and targeted to capture as many/complete relationships as possible in the raw data. DL can be classified into supervised, unsupervised and hybrid DL models according to whether labeled data are required or not, where hybrid models usually refer to the use of unsupervised model results as input data or important auxiliary to supervised models. The predecessor and technical essence of DL is artificial neural networks (ANNs). In 1958, the predecessor of ANN, the Perceptron model, was launched (Rosenblatt, 1958), but it was not of practical value because it was too simple and could only handle linear classification problems, not even solving the XOR problem. Therefore, it does not have practical value but mainly lays the ideological foundation for the later algorithms. Research on neural networks entered a bottleneck until the 1980s, for instance, the back propagation (BP) algorithm for training multilayer neural networks/multilayer perceptrons using sigmoid functions for nonlinear mapping (Rumelhart et al., 1986). Based on the forward propagation of traditional neural networks, the BP algorithm adds a backward propagation process of errors, continuously adjusting the weights and thresholds between neurons until the output error reaches a reduction to within the allowed range or reaches a predetermined number of training times. It effectively solves the problem of nonlinear classification and learning and is the basis for improving and applying neural networks.

However, as the scale of the neural network increases, the BP algorithm suffers from the problem of “gradient disappearance.” Meanwhile, the limited hardware level of computers led to poor computing power, which could not help the further development of BP algorithm, plus the effect of classification and regression application of shallow ML such as SVM in the same period was continuously proved, and DL thus entered the second winter period. Even during the winter period, algorithms such as convolutional neural networks (CNN) and long short-term memory (LSTM) were developed and are still adopted today to process vision tasks (Lecun et al., 1989). Among them, LeNet-5 was proposed by Lecun et al. (1998) and has become the prototype of most deep convolutional neural networks (DCNNs).

Until Hinton and Salakhutdinov (2006) proposed the concept of DL, the problem of “gradient disappearance” was solved, i.e., the algorithm was trained layer by layer by unsupervised learning and then tuned using a supervised back-propagation algorithm. Hinton and his student Alex Krizhevsky used AlexNet to win the ImageNet competition (Smirnov et al., 2013), which became the pioneer of the current wave of deep learning. Its top 5 accuracy rate of 84.6% has an error rate of only 15.3%, and the network is characterized by (1) the use of the ReLU method to speed up training; (2) the use of dropout to prevent overfitting; and (3) GPU parallel computing technology to solve the problem of long optimization time for deep networks with many parameters. Moreover, some neural network architectures, such as variational autoencoders (VAEs) and generative adversarial networks (GANs), have recently attracted much attention in the DL community. The bidirectional encoder representation from transformers (BERT) model proposed by Devlin et al. (2019) has built a transformer network structure with a self-attention mechanism as the core. Excellent performance is presented in many tasks in natural language processing (NLP) due to its versatility. Essentially, DL is a statistical technique with advantages and limitations that are maturing in the areas of computer vision, natural language processing, and speech recognition.

### Reinforcement Learning

Reinforcement learning is a special class of ML algorithms, the most important feature of which is learning from interaction (Keerthi and Ravindran, 1994; Kaelbling et al., 1996). On the basis of interaction, we constantly judge whether the action is related to the goal, corresponding to the generation of rewards or penalties, and repeatedly execute it to finally maximize the expected benefits, an “automatic scoring and escalation” process. Deep reinforcement learning (DRL), a new research hotspot, combines the perceptual capability of deep learning with the decision-making capability of reinforcement learning to achieve direct control from raw input to output through end-to-end learning for applications in robot control, computer vision, natural language processing, and medical care (Erev and Roth, 1998; Frank et al., 2004; Kober et al., 2013; Mnih et al., 2015).

### Evaluation Criteria and Algorithmic Workflows

Different algorithms have their own advantages and disadvantages, and there is no superiority or inferiority. What needs to be done is to fully interpret the input data based on different demand scenarios and then build suitable models to continuously adjust to achieve the best performance. Moreover, the belief that “as long as the most advanced and complex model is used, the scientific problem will be solved” is not objective. In essence, computer technology only assists people in making decisions or automates the human decision-making process and improves efficiency. Therefore, the choice of model should be the most suitable one, rather than pursuing the most complex one. There are four criteria used to judge the merits of machine learning algorithms (Greener et al., 2022). (1) Correctness, the most important criterion for judging the merits of an algorithm. (2) Robustness, i.e., fault tolerance, representing the algorithm’s ability to respond to and address illegal data input. (3) Readability, easy-to-understand algorithms means a less time-consuming process of debugging, modification, and expansion. (4) Temporality, i.e., time complexity and space complexity, represent the computational effort and memory space required to execute the algorithm, respectively.

**Figure 3:**
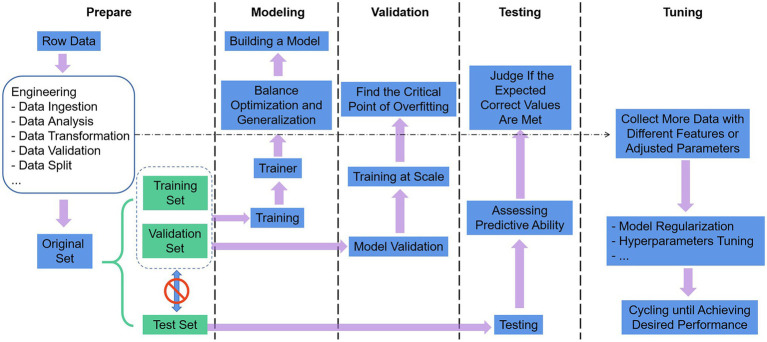
Machine learning workflow.

The use of ML as a technical tool to solve scientific problems can generally comply with the following five steps in Figure 3 (Greener et al., 2022). (1) Define the problem, prepare and process the data, and determine the assessment method. The data were split into three groups: training set, validation set, and test set. The training set is given to build the model, the validation set and the test set both refer to the data samples retained when training the model, and the ability of the model to use the training data should be evaluated successively. The data also undergo targeted preprocessing before use, such as vectorization, value normalization, and feature engineering needed for non-DL. Then, we select the most representative evaluation metrics and validate the evaluation method for the problem. Commonly used performance metrics are confusion matrix, precision, recall, specificity, F1 score, precision-recall curve, ROC, AUC, etc. Common evaluation methods include simple leave-out validation, *k*-fold cross-validation, repeated k-fold validation with disrupted data, and bootstrapping. (2) Build the model. Develop models that are more optimized than the benchmark, with the ultimate goal of balancing the dichotomy between optimization and generalization: find the line between underfitting and overfitting and maximize generalization capabilities. (3) Validating the model. Models with statistical efficacy tend to require scaling up the model first, and a threshold of overfitting for monitoring training losses and validation losses will be required. (4) Testing the model. The goal is to evaluate the predictive capability of the model in completely new data, as opposed to validating the data. It is essential to evaluate all aspects of the model, for instance, to check whether the output of the program meets the expected correct values and whether the model results meet the expected evaluation requirements (accuracy or error). (5) Tuning the model. Boosting the performance of the algorithm with more data, different features, or tuned parameters. The previous steps are repeated continuously, with model regularization and tuning of hyperparameters (parameters to control the behavior of the algorithm when building the model) depending on the performance of the model on the validation set until the desired performance is achieved.

Machine learning methods tend to require a combination of mathematical knowledge concerning statistical probability theory, linear algebra, and algorithmic complexity theory, combined with the diversity of microbial data, which makes it intractable for researchers in the field of microbiology to construct and utilize complex ML models independently. In response to the nature and volume of experimental data specific to various research directions, experts in big data science propose ideas and technical support on approaches to leveraging existing data for effective ML, facilitating the emergence of new cross-cutting areas. With the widespread adoption of ML and DL algorithms, humans have been presented with a whole new world of microorganisms, especially in the fields of classification and prediction.

## Classification and Prediction

Next, we will characterize the impact of ML on the microbiology field and specific application cases. The application of ML in microbial species and community classification and prediction mainly includes microbiome and taxonomy, microbial ecology, pathogen and epidemiology, and drug discovery (with a particular focus on antibiotics/antimicrobial peptides).

### Microbiome and Taxonomy

The microbiome refers to an ecological community of microorganisms with different characteristics and functions that coexist in a given environment, including the genomes and environmental/habitat conditions of the members (Lederberg and McCray, 2001; Berg et al., 2020). The application usually combines one or more of the multiomics techniques, which to some extent is more accurate and precise in classification than single-omics data studies and facilitates the exploration of the influential factors in microbiomics network mechanisms. The strategy of microbial taxonomy is to distribute target sequences to microbial communities at different taxonomic levels utilizing various tools (Marchesi and Ravel, 2015). ML, especially classification and clustering algorithms, allows classification based on data representing the characteristics/functionality of the target community, reflecting similar relationships. With upgrades in sequencing technology, hundreds of millions of short sequencing reads have been generated from merely a single sample, which consequently generates high-dimensional microbiome data (Luz Calle, 2019). Therefore, linear or nonlinear dimensionality reduction algorithms are advantageous in handling complex and multivariate sparse microbiome data to achieve dimensionality reduction (Kostic et al., 2015) and visualization (Song et al., 2019) of the data space.

Common supervised classification algorithms are particularly valuable in identifying highly complex datasets, as in the case of human microbiota surveys (Knights et al., 2011). ML and statistical techniques are in place to build predictive models of taxonomic units (Knights et al., 2011) or functions (White et al., 2009) to distinguish between distinct sample groups. The selection of classification techniques requires considering the characteristics of different microbial communities and extracting the data with different features, encoding the extracted data with labels, and rendering them available for model training (Knights et al., 2011). A study as early as 2012 demonstrated that random forests enable effective and accurate classification of human microbial community samples and allow the identification of key components (OTUs or species) that differentiate between groups (Knights et al., 2011; Yatsunenko et al., 2012). A series of base classifiers are trained separately and independently, and the results of each base classifier training are integrated by adopting a certain rule. This is the idea of ensemble learning, which will obtain better classification results than a single classifier (Wang et al., 2007; Wu and Zhang, 2008). Subsequent studies have targeted the oral microbiota in saliva and classified them with the algorithm of SVM, ANN, and DT (Nakano et al., 2014). Xu et al. (2020) constructed classifiers and classified new samples using LR, SVM and DT based on the dimensionality reduction space generated by t-SNE with Aitchison distance, compared the classification performance of the same classifiers in the original dimension and the dimensionality reduction space, and demonstrated that compared with the t-SNE dimensionality reduction technique using Euclidean distance, Aitchison distance increases the classification accuracy (ACC) of the classifier in general.

Unsupervised learning relies on the strategy of sequencing depth information or OTU clustering of sample data instead of known information (Sangwan et al., 2016). MetaBAT quantifies the similarity of sequences using sequence similarity and information about the sequencing depth of the sample data, using the calculation of the distance between overlapping clusters, and then clustering (Kang et al., 2015). COmposition, read CoverAge, CO-alignment, and paired-end read LinkAge (COCACOLA) calculates the distance with L distance instead of the traditional Euclidean distance (Lu et al., 2017). Strategies for OTU-based clustering inevitably take into account the setting of thresholds, feature extraction, and the choice of specific clustering methods. Cai and Sun (2011) proposed a hierarchical clustering method, i.e., first filtering a large number of unnecessary sequence comparisons with k-mers and then launching the hcluster algorithm in the clustering process to achieve similar accuracy as the standard hierarchical clustering algorithm.

The design of sequence classification methods based on deep learning is not rare. A study proposed a sequence classification technique based on k-mer and two DL architectures—CNN to discriminate and deep belief network (DBN) to generate—for the bacterial taxonomy of macrogenomic data (Fiannaca et al., 2018). The ANN classifier can optimize the classification effectiveness and confidence of the target community after feature analysis. A study developed a pipeline (cell type recognition and CellCognize) based on multidimensional flow cytometry (FCM) data *via* ANN to enable quantification of cell type diversity and subsequent differentiation and classification of microbiota of known composition (Duygan et al., 2020). Composed of a feed-forward back-propagation algorithm, an input layer, a hidden layer, and an output layer, ANNs have been trained to classify either five or 32 standard multiparameter FCM datasets and forecast cell type attribution of FCM data from poorly trained microbial samples of known or unknown composition.

Given the characteristics of high dimensionality, multinoise, data sparsity, and heterogeneity of histological data, as well as the problem of unbalanced datasets in experiments, the integration of complex and large-scale histological data imposes high demands on the analysis capability of algorithmic models and computing platforms. Currently, the main methods are dimensionality reduction and noise reduction through PCA or autoencoder and transformation of sparse and heterogeneous data through regression methods. However, all of these methods have their drawbacks, and a substantial amount of research on these issues will be necessary in the future.

### Microbial Ecology

Microbial ecology, with its origins in environmental microbiome studies, takes as its starting point the study of target microbiota, with the long-term goal of capturing the diversity of multiple species interactions (competition, predation, facilitation, and symbiosis), as well as uncovering their distribution patterns/networks and maintenance mechanisms. Mechanisms of microbiota–microbiota and host–microbiota interactions are critical to our understanding of microbial network structure and function of homeostasis in a given habitat (Broberg et al., 2018; Hassani et al., 2018; Van De Guchte et al., 2018). Advances and applications of new experimental and computational methods will drive the integration of microbial ecology research with leading technologies in integrated multiomics, computational biology, and other fields.

The purpose of constructing ecological networks is to model all interactions between species and their environment. Faisal et al. (2010) used four widely used statistical/ML methods, graphical Gaussian models (GGMs), L1-regularized regression with the least absolute shrinkage and selection operator (LASSO), sparse Bayesian regression (SBR), and Bayesian networks (BNs), to validate their usefulness in identifying community interactions in microecological networks. These methods enable simulated restoration of food web structure and contribute to modeling and predicting the effects of bioclimatic variables. However, since the complete ecological knowledge of the true interaction network between species is hardly visible, assessing the success of the modeling solely relies on known or possible relationships. Although pairwise interactions are the basis for the study of complex ecological networks, higher-order interactions involving three or more taxonomic units increase the variability and stochasticity in the study of community composition, on which the prediction of microbiota-associated biological phenotypes is based (De’ath and Fabricius, 2000).

It is necessary to simplify scientific problems by switching predictive strategies based on species characteristics when predicting relationships (natural diversity, life cycles, interactions, and coevolution) across species or with their environment. Leite et al. (2018) explored several machine learning techniques (kNN, RF, SVM, and ANN) to predict/identify the presence of a given phage–bacterial pair interaction after 10-fold cross-validation based on accuracy, F-score, specificity, and sensitivity criteria to filter the most predictive algorithms and their parameter values. The theoretical basis of its prediction lays in the interaction between a given phage–bacterial pair of encoded proteins, allowing the work to be converted into protein–protein interaction (PPI) prediction (Cusick et al., 2009). Accordingly, two features, the domain–domain interaction score and protein-level structural information, were selected in the feature extraction phase.

The intersection of genetics and ecology is established on the basis of the population concept. Stupp et al. (2021) proposed supervised ML-based phylogenetic profiling (MLPP) to predict functional interactions between human genes and the interaction environment in which they occur (i.e., biological functions) and established a web server containing functional interaction predictions for all human genes. They predicted the probability of all possible gene pairs in each tag using the lightGBM model, which is related to RF after comparing it with the DT, LR, and NB algorithms. Based on simulations and real data, Pichler et al. (2020) compared generalized linear models (GLM) with ML models (RF, boosted regression trees, deep neural networks, CNN, SVM, NB, and kNN) to measure their capability to predict species interactions based on traits and to extrapolate trait combinations that are causally relevant to species interactions. In a global crop–pollination database, they found that RF had the best predictive performance, predicting species interactions in plant-pollinator networks remarkably well.

However, the reality that most microbial species within communities are not culturable makes the prediction of interspecies interactions in natural microbial communities challenging. This comes from the fact that the accuracy of deep learning (especially deep neural networks) depends on the reliability of the training database. Moreover, there is still space for investigators to design joint experimental and modeling studies to uncover interaction mechanisms that have not yet been fully investigated (Lee et al., 2020).

### Pathogen and Epidemiology

Epidemics have the characteristics of being contagious, epidemic, and recurrent. Infestations of previously unknown pathogenic microorganisms pose a continuous threat to food security and human health. To address the medical challenges of epidemiology, the identification, and characterization of pathogens, and the screening and prediction of diseases have emerged as major concerns for professional biomedical scientists. ML, as well as DL, which dominates in batch image classification, has led to a significant reduction in the time and computational cost spent on dataset analysis due to its extremely efficient, cost-effective, accurate and high-throughput advantages (Ghosh et al., 2022).

Disease epidemiology studies examine the patterns of temporal and spatial dynamics of diseases at the population level under different environmental conditions. Research on issues such as diseases caused by plant and animal viruses provides a large dataset on gene expression, chromosome conformation, genetic variation, traits, and diseases. The relevance of the viral genome allows for screening with the help of macrogenomics. The application of ML enables the integration of multiomics data and significantly improves macrogenomic data analysis. ML assists in classifying these viral sequences and thus deepens our understanding of virus evolution. VirFinder is a k-mer-based platform to identify prokaryotic virus sequences from mixed macrogenomic data, accelerating the screening of pathogens at the genetic level in plant and animal virome studies (Ren et al., 2017). The synergistic application of ML and hyperspectral imaging (HSI) provides a new methodological idea for image detection of viral diseases. While the high-dimensional data generated by HSI contain redundant information, ML reduces the dimensionality of HSI data by determining the effective specific wavelength range through data preprocessing. For instance, multilayer perceptrons (MLPs), ANNs, and CNNs enable the detection and classification of color images by hidden image features with high accuracy of >96.00% (Lowe et al., 2017). Compared to traditional ML, which requires feature extraction techniques tailored to the nature of the data and model, DL supports automatic feature extraction, reducing computational time, and the burden of reliance on professional expertise. Training a model (classifier) with live images is a case in point (Ferentinos, 2018). This implies that determining the reliability of the classification relies to some extent on the abundance of available images based on the scene. For example, VGGNet, obtained by Chen et al. (2020), achieved an average accuracy of 92.00% for predicting rice plant image categories based on ImageNet and Inception module pre-training.

Phages are the most abundant organisms on Earth and have been considered as natural enemies of bacteria. Several ML algorithm models aiming to improve the automatic recovery and prediction of phages already exist. For instance, VirSorter searches the database of expected proteins up front using probabilistic similarity and reference homology to identify viral signals, but the disadvantage is that it does not fully distinguish between virus and nonvirus Pfam annotations (Roux et al., 2015). Kaelin et al. (2022) employed VirSorter v.1.0.5 to identify circular contigs of candidate viruses. Another tool, Meta-genomic Analysis and Retrieval of Viral Elements (MARVEL), which aggregates annotation and sequence signature information of previously identified phages, was developed to identify and predict double-stranded DNA phage sequences in macrogenomic boxes. Given the excellent recall, Braga et al. (2020) used MARVEL to identify phage bins for prediction. According to the authors’ statement, comparing the performance of MARVEL, VirFinder, and VirSorter, all three performed comparably in terms of specificity, with MARVEL outperforming in terms of sensitivity (Amgarten et al., 2018). VIBRANT, the first computational tool to utilize neural networks and protein similarity methods, had a particularly strong performance score (94%) in the automatic recovery of microbial viruses, which was stronger than the first three (Kieft et al., 2020). Luo et al. (2022) filtered ≥ 1 kb contigs to identify viral contigs and related reads *via* VIBRANT. We summarize the available data and materials, which are shown in Table 1.

**Table 1:** The available data and materials for prediction of pathogens and epidemiology.

Tools	Availability of data and materials	References
VirSorter	https://github.com/simroux/VirSorter	Roux et al., 2015
VirSorter2	https://bitbucket.org/MAVERICLab/VirSorter2	Guo et al., 2021
VirFinder	https://github.com/jessieren/VirFinder	Ren et al., 2017
DeepVirFinder	https://github.com/jessieren/DeepVirFinder	Ren et al., 2020
MARVEL	https://github.com/LaboratorioBioinformatica/MARVEL	Amgarten et al., 2018
VIBRANT	https://github.com/AnantharamanLab/VIBRANT/	Kieft et al., 2020

To date, most of the results generated from the intersection of pathogen research and machine learning in epidemiology have been prospective and feasible. Comparing different stages of classifier innovation, we found that feature extraction and ranking that include multiple layers of information enhance the prediction accuracy of the model. The embedding of DL refreshes our knowledge of pathogen features.

### Drug Discovery (With a Particular Focus on Antibiotics/Antimicrobial Peptides)

The abuse of antibiotics has led to a worsening problem of drug resistance in pathogenic bacteria, which has been an enormous threat to human health. Screening for secondary metabolites in soil microorganisms that inhibit the growth of pathogenic bacteria is regarded as the traditional primary means of antibiotic discovery (Wright, 2017). The current dilemma of decreasing the rate of discovery of new antibiotics urgently needs to be addressed. In addition, the administrative costs of screening approaches based on large synthetic chemical libraries and the high rate of antibiotic design attrition have increased the necessity for new antibiotic discovery methods to improve the rate of new antibiotic discovery. Modern drug discovery has entered the era of big data. AI modeling of the dynamic, heterogeneous, and large-scale nature of drug datasets continues to drive paradigm innovation in drug discovery (Zhu, 2020).

Techniques to identify and predict new antibiotic structural classes with the help of ML are largely mature and widely adopted (Camacho et al., 2018). DL accelerates the screening process of compounds with antibiotic properties from existing chemical libraries (Dimasi et al., 2016). Antimicrobial peptides (AMPs) are candidates for coping with antibiotic resistance. Researchers have successively established several antimicrobial peptide databases containing data on various types of AMPs from various sources, such as APD, CAMP, and AVPDB, which greatly facilitate mining and forecasting of AMPs. Fu et al. (2020) designed a DL model for high-throughput antibacterial peptide recognition (ACEP), which is innovative in that it introduces an amino acid embedding tensor to capture the similarity between amino acids, constructed a “convolution and concatenation” (CVCA) layer using the attention mechanism of natural language processing (NLP) to fuse various heterogeneous information or features, and quantified the contribution of different components of the model to the final prediction using the attention scores of different parts of the peptide sequence. Capecchi et al. (2021) trained recurrent neural networks (RNNs) using sequence information from DBAASP v.2 (Database of Antimicrobial Activity and Structure of Peptides, now updated to DBAASP v.3; Pirtskhalava et al., 2021), including AMP and non-AMP datasets, and hemolytic and non-hemolytic data, mixing the use of supervised and unsupervised learning for the first time, maximizing the utilization of highly selected posterior data. The study also synthesized and tested 28 sequences generated and selected, yielding 12 new active AMPs, eight of which were also non-hemolytic. Das et al. (2021) designed a fully automated computational framework for molecular targeting and screening, in which conditional latent (attribute) space sampling (CLaSS) was designed for target generation, which is more efficient and easily reusable than other ML methods. The framework generates a potential space of molecular information *via* deep generative autoencoder modeling, utilizes a classifier for training guidance, and filters the generated molecules through deep learning classifiers based on the physicochemical features obtained in high-throughput molecular dynamics simulations. This study reported 20 CLaSS-generated AMP sequences and 11 non-AMP sequences obtained *via* the above screening method, which was shown to be less prone to false negatives. Wang (2022) combined various NLP neural network models (NNMs), built a pipeline containing LSTM, attention, and BERT, and established a DL method that adapts to learn AMP sequence features to mine and identify novel AMPs. Among a total of 2,349 sequences identified as candidate AMPs, 216 were chemically synthesized, including 181 indicative of antibacterial activity (>83% positivity). The code availability is shown in Table 2.

**Table 2:** The code availability for prediction of antimicrobial peptide (AMP) discovery.

Tools	Code availability	References
ACEP	https://github.com/Fuhaoyi/ACEP	Fu et al., 2020
RNN	https://github.com/reymond-group/MLpeptide	Capecchi et al., 2021
CLaSS	https://github.com/IBM/controlled-peptide-generation	Das et al., 2021
AMP prediction pipeline with NNMs	https://github.com/mayuefine/c_AMPs-prediction	Wang, 2022

Overall, the time is ripe for modern ML/DL applications for antibiotic discovery (Cardoso et al., 2020). Their effective contribution to the bulk filtering and prediction of antimicrobial peptides is alleviating concerns about the high risks and low returns associated with antibiotic development. Notably, the high success rate of deep neural network model-guided antibiotic development is heavily dependent on the combination of model prediction and appropriate experimental design, and this wet-dry combination strategy is a scientific idea that has started to be popularized after the discovery of complementary information and experimental practices.

## Conclusion

Research in machine learning and deep learning is evolving rapidly, with architectures, algorithm combinations, and computational strategies changing rapidly. The ultimate goal is not only to predict the accuracy of the task but also to uncover the underlying biological processes in the scientific problem. The perception that “deep learning may eventually eliminate all other machine learning algorithms” is limited and one-sided. Deep learning modeling requires a large amount of training data to demonstrate fantastic performance, but realistic colony research frequently encounters problems with small sample datasets. At this point, deep learning methods fail to attack them, but traditional machine learning methods are capable of handling them. The development of effective analytical tools, including software for data mining and machine learning, ensures data validity, proper annotation, and open sharing, allowing most studies arising from the intersection of microbiology and machine learning to show promising findings. After bioinformatics and multiomics integration, ML and DL will lead the next wave of technologies to uncover biological regularity.

## Author Contributions

YJ drafted the manuscript. JL and DH modified the English content of this manuscript. YL and D-dL conceived the idea. All authors contributed to the article and approved the submitted version.

## Funding

The work was financially supported by the China Postdoctoral Science Foundation (2021M701987; D-dL) and (2021M700084; YL).

## Conflict of Interest

The authors declare that the research was conducted in the absence of any commercial or financial relationships that could be construed as a potential conflict of interest.

## Publisher’s Note

All claims expressed in this article are solely those of the authors and do not necessarily represent those of their affiliated organizations, or those of the publisher, the editors and the reviewers. Any product that may be evaluated in this article, or claim that may be made by its manufacturer, is not guaranteed or endorsed by the publisher.
